# A Systematic Review of the Extra-Hepatic Manifestations of Hepatitis E Virus Infection

**DOI:** 10.3390/medsci8010009

**Published:** 2020-02-04

**Authors:** Prashanth Rawla, Jeffrey Pradeep Raj, Alan Jose Kannemkuzhiyil, John Sukumar Aluru, Krishna Chaitanya Thandra, Mahesh Gajendran

**Affiliations:** 1 Department of Medicine, Sovah Health, Martinsville, VA 24112, USA; 2 Department of Clinical Pharmacology, Seth G.S. Medical College & King Edward Memorial Hospital, Mumbai 400012, India; jpraj.m07@gmail.com; 3 St. Johns Medical College, St. John’s National Academy of Health Sciences, Bengaluru, Karnataka 560034, India; alanjosekannemkuzhiyil@gmail.com; 4 Beth Israel Deaconess Medical Center, Harvard Medical School, Boston, MA 02212, USA; Jaluru@bidmc.harvard.edu; 5 Department of Pulmonary and Critical Care Medicine, Sentara Virginia Beach General Hospital, Virginia Beach, VA 23454, USA; kc_thandra@yahoo.com; 6 Department of Internal Medicine, Texas Tech University Health Sciences Center El Paso, Paul L. Foster School of Medicine, El Paso, TX 79905, USA; mahesh.gajendran@ttuhsc.edu

**Keywords:** hepatitis E, HEV, viral hepatitis, extra-hepatic manifestations

## Abstract

Hepatitis E virus (HEV) is a non-enveloped, positive-sense, single-stranded RNA icosahedral virus belongs to the genus *Orthohepevirus* within the Hepeviridae family. HEV infection can be asymptomatic, or it can cause icteric or fulminant hepatitis. Off late, there have been a number of publications reporting the extra-hepatic manifestations of HEV infection, and this systematic review is aimed at summarizing the available evidence in this regard. Two independent investigators searched PubMed, PubMed Central and Embase databases using the search string “(((hepatitis E) AND (Extrahepatic OR Extra-Hepatic))) OR ((Hepatitis E) AND (Neurology OR Cardiology OR Respiratory OR Lung OR Gastrointestinal OR musculoskeletal OR immunology OR pulmonary)) Filters: Abstract availability, English language, and Human studies”. The extra-hepatic manifestations reported in each of the selected articles were classified and reported as neurological, cardiovascular, and hematological and miscellaneous manifestations. The total number of various manifestations reported in our study were *n* = 324. These include neurological manifestations (*n* = 178/324 (54.94%)), cardiovascular and hematological manifestations (*n* = 113/324 (34.88%)), gastro-intestinal/pancreaticobiliary manifestations (*n* = 24/324 (7.41%)) and other rarer manifestations involving systems such as renal (*n* = 4/324; 1.24%), endocrine (*n* = 1/324; 0.31%), dermatology (*n* = 1/324; 0.31%), respiratory (*n* = 1/324; 0.31%), muscular (*n* = 1/324; 0.31%) and immune system (*n* = 1/324; 0.31%). Thus, HEV can have extra-hepatic manifestations affecting any system of the human body. Further research is needed to elucidate the underlying pathophysiological manifestations of these extra-hepatic manifestations and to prove causal association with HEV.

## 1. Introduction

Hepatitis E virus (HEV) is a non-enveloped, positive-sense, single-stranded RNA icosahedral virus that belongs to the *Orthohepevirus* within the Hepeviridae family. Transmission of HEV can occur through the fecal-oral route by contaminated food and water, transfusion of blood, and through mother-to-child vertical transmission. Although person-to-person transmission is uncommon, patients are infectious during fecal shedding [1]. HEV is represented by one serotype and four main genotypes wherein genotypes 1 and 2 spread via the fecal–oral route and cause the epidemics, while genotypes 3 and 4 are zoonotic [2]. Genotypes 1 and 2 are endemic in many developing nations. Genotype 1 is the most common cause of acute hepatitis in Asian countries, especially in India. Genotype 2 is seen commonly in Africa and Central America, Genotype 3 is prevalent in western countries, as well as in Asia and North America, while genotype 4 is detected in Asian and European countries [3]. HEV infection is one of the most common causes of acute hepatitis globally with 20 million infections annually and an estimated 70,000 deaths attributed to genotypes 1 and 2 [4].

HEV infection can be asymptomatic, or it can cause icteric or fulminant hepatitis [5]. The seroprevalence of HEV was estimated at 6% in the USA, 11% in the UK, and 52% in the hyperendemic Toulouse region of south-west France in 2015 [6]. A more recent meta-analysis of 2018 has shown that prevalence is up to 9% in the USA, 4.2% in Brazil and up to 1% in the Mixed Caribbean [7]. Chronic HEV infection has also been reported, commonly associated with genotype 3, resulting in progressive liver failure, liver fibrosis, and cirrhosis [8]. Of late, there have been a number of publications reporting the extra-hepatic manifestations of HEV infection, both acute and chronic, mainly due to the temporal association between the infection and the extra-hepatic manifestations after excluding the other possible etiologies that may mimic the manifestations. These extra-hepatic manifestations may thus distract the physician from diagnosing HEV infection, and this systematic review is aimed at summarizing the available evidence in this regard.

## 2. Methods

Two independent investigators (JPR and AJK) searched PubMed, PubMed Central and Embase databases on 20th October 2018. The search string used in these databases were “(((hepatitis E) AND (Extrahepatic OR Extra-Hepatic))) OR ((Hepatitis E) AND (Neurology OR Cardiology OR Respiratory OR Lung OR Gastrointestinal OR musculoskeletal OR immunology OR pulmonary)) Filters: Abstract availability, English language, and Human studies”. Discrepancies between the two authors were adjudicated by another author (PRR). Review articles, letters to editors where abstract was not available and articles in whom the full text was not available were excluded. Finally, the extra-hepatic manifestations reported in each of the selected articles were classified and reported as neurological, cardiovascular, and hematological and miscellaneous manifestations.

## 3. Results

### 3.1. Demographics

**Figure 1:**
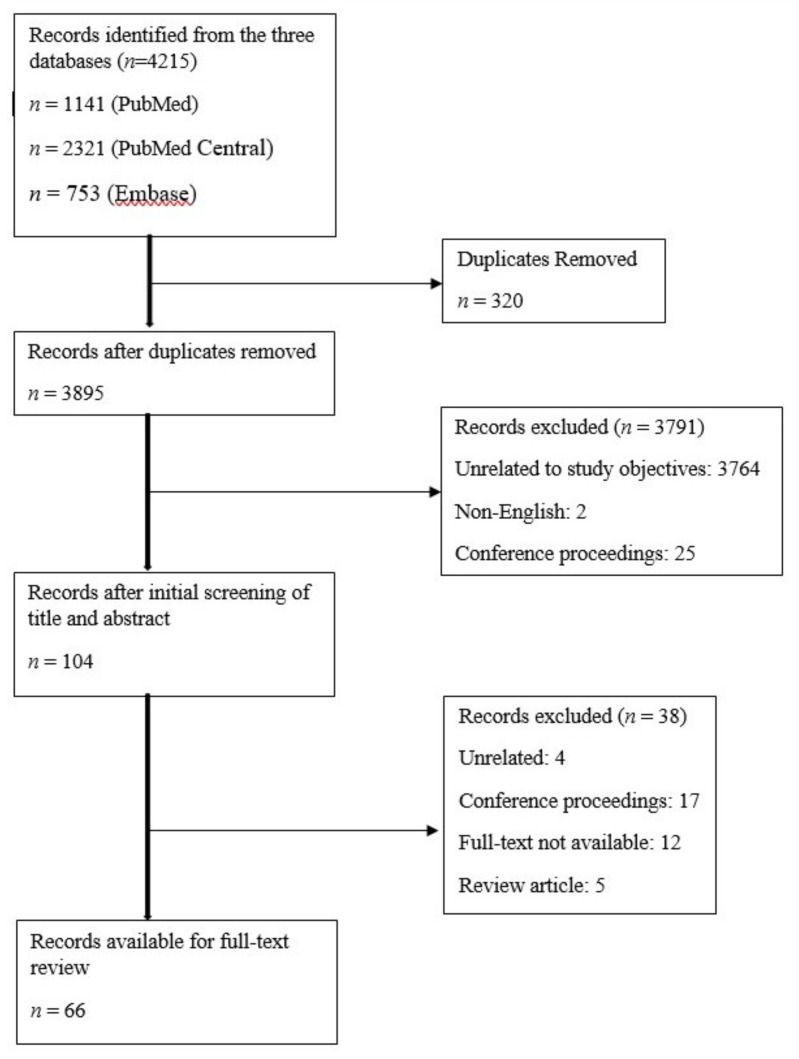
PRISMA diagram.

A total of 4215 articles (*n* = 1141 in PubMed; *n* = 2321 in PubMed Central and *n* = 753 in Embase) were retrieved based on the search strategy. After removing duplicates, articles unrelated to the objective, conference proceedings, unavailable full-texts, and non-English reports, the final number of articles included in this systematic review was *n* = 66. The PRISMA (preferred reporting items for systematic reviews and meta-analyses) flow diagram is given in Figure 1.

The total number of various manifestations reported in our study were *N* = 324. Neurological disorder was the most common extrahepatic manifestation of hepatitis E infection, with a total of *n* = 178/324 (54.94%) manifestations having been reported in our review. This was followed by the cardiovascular and hematological manifestations that accounted for *n* = 113/324 (34.88%) manifestations. The third common system involvement was the gastro-intestinal/pancreaticobiliary system that accounted for *n* = 24/324 (7.41%) manifestations. Rarer manifestations involving systems such as renal (*n* = 4/324; 1.24%), Endocrine (*n* = 1/324; 0.31%), Dermatology (*n* = 1/324; 0.31%), respiratory (*n* = 1/324; 0.31%), muscular (*n* = 1/324; 0.31%) and immune system (*n* = 1/324; 0.31%) were also reported.

### 3.2. Neurological and Musculoskeletal Manifestations

Among the neuro-muscular manifestations, the commonly reported were neuralgic amyotrophy (*n* = 102/179; 56.98%) [9,10,11,12,13,14,15,16,17,18,19,20,21,22,23,24] and Guillain–Barré syndrome (*n* = 36/179; 20.11%) [10,24,25,26,27,28,29,30,31,32,33,34,35,36,37,38,39,40]. The other rarer neurological manifestations that were reported include mononeuritis multiplex [10], encephalitis [11,13,39,41], cerebral ischemia [11,39], myasthenia gravis [42], polyneuromyopathy [24,43], meningo-radiculitis [10,44], epilepsy [11], encephalopathy [45], facial nerve palsy [11,46,47], encephalitic parkinsonism [48], transverse myelitis [49], peripheral neuropathy [24,39,50], vestibular neuritis [24], small fiber neuropathy [24], Myositis [51], and certain non-specific neurological symptoms like myalgias, joint pains, etc. [52,53] which are summarized in Table 1. 

### 3.3. Cardiovascular and Hematological Manifestations

Among the cardiovascular and hematological manifestations, the commonly reported ones include cryoglobulinemia (*n* = 51/113; 45.13%) [55] and monoclonal gammopathy (*n* = 17/113; 15.04%). [24] The cardiovascular manifestations include myocarditis [56,57], cardiac arrhythmias [24], long QT syndrome [58], and Torsade’s de pointes [58]. The other hematological manifestations that were reported include anemia [59], thrombocytopenia, [24,59] lymphocytosis, [24] lymphopenia [24], leukocytosis [60], massive hemolysis [61], metabolic acidosis [60], and hematological malignancies [24] which are summarized in Table 2.

### 3.4. Gastrointestinal/Pancreatico-Biliary and Miscellaneous Manifestations

With regards to the gastro-intestinal/pancreaticobiliary system, pancreatitis (*n* = 22/24; 91.67%) [60,62,63,64,65,66,67] was the most common manifestation. One of these patients also had a pancreatic pseudocyst [66]. The other manifestation reported from this system was acalculous cholecystitis [68]. Table 3 summarizes the reports of manifestations of other systems such as endocrinology [69], dermatology [70], immunology [71], renal [60,61] and respiratory [72] whose frequencies of occurrence have been rare.

## 4. Discussion

In this systematic review, we have summarized the frequencies of various neurological, cardiovascular, hematological, gastrointestinal and other systemic extra-hepatic manifestations of HEV infection that have been reported in the literature. We report that neurological manifestations are more common over the other manifestations, which are followed by the hematological manifestations. However, it is clear that any body system could be affected by HEV and can present with manifestations. HEV infection is a self-limiting illness lasting a few days with symptoms of hepatitis such as jaundice, fever, nausea, vomiting, abdominal pain, anorexia, and malaise [75]. Mortality is greater in patients who are pregnant or have chronic liver disease [75]. However, chronic infection has also been reported especially with genotype-3 [75]. 

Various extra-hepatic manifestations of HEV have been reported from time to time, and the neurological manifestations clearly take the lead. The acute inflammatory demyelinating polyradiculoneuropathy (AIDP) is the most common variant of HEV associated GBS (Guillain-Barre syndrome) [76]. Whereas, in a vast majority of patients with neuralgic amyotrophy, the brachial plexus or the phrenic nerve was most commonly affected. These patients are thought to be genetically predisposed for an autoimmune response [77] Though the pathophysiology behind these manifestations is largely unclear, three hypotheses have been put forth [75,76,77], the first hypothesis being the presence of HEV quasi-species with the ability to infect neurologic tissues [75,77]. This is supported by the fact that HEV RNA had been isolated from the cerebrospinal fluid (CSF) of patients, and it was found to be different from that of the ones seen in the serum at the same time point, suggesting the presence of certain neurotropic variants [52]. However, this has not always been the case wherein HEV RNA has not been isolated from the CSF [38]. The second hypothesis being the ability of the virus to acquire host RNA sequences, thereby the property to infect multiple cell types including the CNS [77]. The third one being the property of some strains to produce certain antigens which in turn leads to autoimmune inflammatory polyneuropathy via molecular mimicry [76,77]. In patients with neurological disorders especially peripheral nerve involvement and concurrent liver enzyme elevation it is recommended that clinicians consider the possibility of HEV infection.

The most striking hematological manifestation is cryoglobulinemia, and the pathophysiology is largely unknown. One hypothesis is that there may be an increased expression of serum B cell-activating factor (BAFF), a tumor necrosis factor (TNF)-α family member required for B cell survival that has been documented in certain autoimmune diseases and hepatitis C infection. Thus, cryoglobulinemia has been reported in conjunction with other autoimmune or inflammatory disorders [78], especially in several other viral hepatitis including hepatitis C virus [79]. Thus, for the association between cryoglobulinemia and HEV, it is possible that either HEV causes cryoglobulinemia whose mechanism is currently unknown, or pre-existing cryoglobulinemia weakens the immune system of the patient who subsequently acquires HEV infection [24]. The same could be true for the hematological malignancies, as well [79]. Cryoglobulinemia, in turn, can cause renal injury and glomerulonephritis [80]. Though thrombocytopenia could be due to autoimmune causes as in other viral infections [81]. the changes in lymphocyte and platelet counts are most likely to be non-specific, found following inflammatory stimuli as these changes had no clinical consequences [24].

With regards to pancreatitis, it was once thought to be mostly associated with fulminant hepatitis, but of late, this dictum has been proven to be wrong as more and more pancreatitis has been reported in patients without fulminant hepatitis [81]. Like other extrahepatic manifestations, the underlying mechanism is largely unknown, and there are a few hypotheses. Some of these include biliary sludging in the early phase of viral illness, the development of edema of the hepatopancreatic ampulla (ampulla of Vater) and a resultant obstruction to the pancreatic fluid outflow, direct inflammation and destruction of pancreatic acinar cell by the virus, release of lysosomal enzymes from the inflamed liver along with the activation of trypsinogen to trypsin and rarely, intrapancreatic hemorrhage due to hypoprothrombinemia or disseminated intravascular coagulation resulting in pancreatic damage and subsequent pancreatitis [81]. Thus, any patient with HEV and abdominal pain out of proportion should be evaluated for acute pancreatitis and conservatively managed [81]. We have also reported a large number of other manifestations, which are much rarer and thus would require more case studies/reports and further research to prove their association with HEV.

Our study, however, has a few limitations. Our main limitation is that this is a review of studies indexed in just three databases and there is a likelihood that we have missed information that was published in non-indexed journals/conference proceedings.

## 5. Conclusions

In this systematic review, we have made an attempt to summarize the plethora of extra-hepatic manifestations of HEV that have been reported as either case reports, case series, retrospective/prospective observational studies. These manifestations could affect any of the systems in our human body. We report that neurological manifestations (GBS or neuralgic amyotrophy) are the most common extrahepatic manifestations followed by cryoglobulinemia and other inconsequential hematological changes. Acute pancreatitis like in any other viral hepatitis is seen associated with HEV also. Further research is needed to elucidate the underlying pathophysiological manifestations of these extra-hepatic manifestations and to prove causal association with HEV.

## Figures and Tables

**Table 1:** Extra-hepatic neurological and musculoskeletal manifestations of hepatitis E infection.

S. No	Disorder	First Author (Year, Study Design)	Number of Patients
1	Guillain–Barré syndrome	Al-Saffar (2018, CR) [25], Bandyopadhyay (2015, CR) [26], Blasco-Perin * (2015, RS) [10], Choudhary (2019, CR) [27], Cronin (2011, CR) [28], Fukae * (2016, CC) [29], Higuchi (2015, CR) [30], Ji (2016, CR) [54], Lei (2017, CR) [31], Loly (2009, CR) [32], Maurissen (2012, CR) [33], Salim (2017, CR) [34], Santos (2012, CR) [35], Scharn (2014, CR) [36], Stevens (2015, RS) [37], Van Den Berg (2014, CC) [38], Wang (2018, CC) [39], Woolson (2014, RS) [24], Zheng (2018, CR) [40].	36
2	Neuralgic amyotrophy (Parsonage Turner syndrome/Brachial neuritis)	Avila (2016, CR) [9], Blasco-Perin (2015, RS) [10], Dalton (2017, CSS) [11]. Dartevel (2015, CS) [12], Deroux (2014, CS) [13], Fong (2009, CR) [14], Frtiz (2018, CC) [15], Jones (2017, RS) [16], Sanchez-Azofra (2018, CR) [17], Scanvion (2017, CR) [18], Silva (2016, CR) [19], Swinnen ^#^ (2018, CR) [20], Theochari (2015, CR) [21], Van Eijk (2014, Cohort) [22], Velay (2017, CR) [23], Woolson (2014, RS) [24].	102
3	Myasthenia gravis	Belbeizer (2014, CR) [42]	1
4	Polyneuromyopathy	Belliere (2017, CR), [43] Woolson (2014, RS) [24]	2
5	Mononeuritis multiplex	Blasco-Perin (2015, RS) [10]	6
6	Meningo-radiculitis	Blasco-Perin (2015, RS), [10] Despierres (2011, CS) [44]	5
7	Non-specific symptoms	Blayney (2018, CR), [53] Kamar (2010, CR) [52]	2
8	Cerebral ischemia	Dalton (2017, CSS), [11] Wang (2018, CC) [39]	5
9	Epilepsy	Dalton (2017, CSS) [11]	2
10	Encephalitis	Dalton (2017, CSS) [11], Deroux (2014, CS) [13], Murkey (2017, CR) [41], Wang (2018, CC) [39]	5
11	Facial nerve palsy	Dalton (2017, CSS) [11], Jha (2012, CR) [46], Yazaki (2015, CS) [47]	3
12	Encephalopathy	De Vries (2014, CR) [45]	1
13	Encephalitic Parkinsonism	Pasha (2018, CR) [48]	1
14	Transverse myelitis	Sarkar (2015, CR) [49]	1
15	Peripheral neuropathy	Bennet (2015, CR) [50], Wang (2018, CC) [39], Woolson (2014, RS) [24]	3
16	Vestibular neuritis	Woolson (2014, RS) [24]	1
17	Small fiber neuropathy	Woolson (2014, RS) [24]	1
18	Myositis	Mengel (2016, CR) [51]	1

CR: case report; RS: retrospective study; CC: Case-Control study; CS: Case Series; CSS: cross-sectional study; * one patient each was reported to have Miller-Fiser variant of Guillain–Barré syndrome; ^#^ report of a patient with anterior interosseous mono-neuropathy.

**Table 2:** Extra-hepatic cardiovascular and hematological manifestations of hepatitis E infection.

S. No	Disorder	First Author (Year, Study Design)	Number of Patients
1	Cardiac Arrhythmia	Woolson (2014, RS) [24]	1
2	Long QT Syndrome and Torsade’s de pointes	Aiqin (2012, CR) [58]	1
3	Myocarditis	Dougherty (2016, CR) [57], Premkumar (2015, CR) [56]	2
4	Anemia	Kishore (2009, CR) [59]	1
5	Thrombocytopenia	Kishore (2009, CR) [58], Woolson (2014, RS) [24]	13
6	Lymphocytosis	Woolson (2014, RS) [24]	14
7	Lymphopenia	Woolson (2014, RS) [24]	8
8	Leukocytosis	Saarwaani (2017, CR) [60]	1
9	Massive hemolysis	Karki (2016, CR) [61]	1
10	Monoclonal gammopathy	Woolson (2014, RS) [24]	17
11	Cryoglobulinemia	Marion (2018, CC) [55]	51
12	Metabolic acidosis	Saarwaani (2017, CR) [60]	1
13	Malignancies (Acute myeloid leukemia, plasmacytoma)	Woolson (2014, RS) [24]	2

CR: case report; RS: retrospective study; CC: case-control study.

**Table 3:** Miscellaneous extra-hepatic manifestations of hepatitis E infection.

S. No	Disorder	System	First Author (Year, Study Design)	No. of Patients
1	Autoimmune Thyroiditis	Endocrine	Dumoulin (2012, CR) [69]	1
2	Cutaneous T-Cell lymphoproliferative disorder	Dermatology	Mallet (2017, CR) [70]	1
3	Pancreatitis	Gastro-intestinal	Deniel (2011, CR) [62], Mithun (2015, RS) [63], Peter (2017, CR) [64], Saarwaani (2017, CR) [60], Sinha (2003, CS) [65], Somani (2009, CR) [66], Thakur (2017, CR) [67]	22
4	Pancreatic pseudocyst	Gastro-intestinal	Somani (2009, CR) [66]	1
5	Acalculous cholecystitis	Gastro-intestinal	Fujioka (2016, CR) [68]	1
6	Acute Graft Dysfunction	Immunology	Hillebrandt (2018, CR) [71]	1
7	Acute Kidney Injury	Renal	Karki (2016, CR) [61], Saarwaani (2017, CR) [60]	2
8	cryoglobulinemic membranoproliferative glomerulonephritis	Renal	Del Bello (2015, CR) [72], Guinault (2016, CR) [73]	2
9	Pleural Effusion	Respiratory	Kumar (2012, CR) [74]	1

No. of: Number of; CR: case report; RS: retrospective study; CS: case series.
